# Peer-led pediatric resuscitation training: effects on self-efficacy and skill performance

**DOI:** 10.1186/s12909-020-02359-z

**Published:** 2020-11-13

**Authors:** M. Binkhorst, J M Th Draaisma, Y. Benthem, E. M. R. van de Pol, M. Hogeveen, E. C. T. H. Tan

**Affiliations:** 1 Radboud Institute for Health Sciences (RIHS), Department of Neonatology, Radboud University Medical Center Amalia Children’s Hospital, P.O. Box 9101, 6500 HB Nijmegen, The Netherlands; 2 Department of Pediatrics, Radboud University Medical Center Amalia Children’s Hospital, Nijmegen, The Netherlands; 3 Department of General Surgery, Radboud University Medical Center, Nijmegen, The Netherlands

**Keywords:** Peer-led learning, Cardiopulmonary resuscitation, Pediatrics, Self-efficacy, Skill performance

## Abstract

**Background:**

Peer-led basic life support training in medical school may be an effective and valued way of teaching medical students, yet no research has been conducted to evaluate the effect on the self-efficacy of medical students. High self-efficacy stimulates healthcare professionals to initiate and continue basic life support despite challenges.

**Methods:**

A randomized controlled trial, in which medical students received pediatric basic life support (PBLS) training, provided by either near-peer instructors or expert instructors. The students were randomly assigned to the near-peer instructor group (*n* = 105) or expert instructor group (*n* = 108). All students received two hours of PBLS training in groups of approximately 15 students. Directly after this training, self-efficacy was assessed with a newly developed questionnaire, based on a validated scoring tool. A week after each training session, students performed a practical PBLS exam and completed another questionnaire to evaluate skill performance and self-efficacy, respectively.

**Results:**

Students trained by near-peers scored significantly higher on self-efficacy regarding all aspects of PBLS. Theoretical education and instructor feedback were equally valued in both groups. The scores for the practical PBLS exam and the percentage of students passing the exam were similar in both groups.

**Conclusions:**

Our findings point towards the fact that near-peer-trained medical students can develop a higher level of PBLS-related self-efficacy than expert-trained students, with comparable PBLS skills in both training groups. The exact relationship between peer teaching and self-efficacy and between self-efficacy and the quality of real-life pediatric resuscitation should be further explored.

**Trial registration:**

ISRCTN, ISRCTN69038759. Registered December 12th, 2019 – Retrospectively registered.

## Background

Pediatric out-of-hospital cardiac arrest is a rare but serious event, with an estimated incidence of 1–20 per 100,000 person-years and a survival rate of 10% or less [1–3]. It has been reported that bystander cardiopulmonary resuscitation (CPR) is provided in only one-third to two-thirds of cases, and that the use of an automated external defibrillator is rare [1, 3]. Proper pediatric basic life support (PBLS) training may reduce the number of casualties.

Healthcare professionals are first and foremost expected to perform high-quality (pediatric) basic life support. This group includes medical students, since society expects them to be equally capable as physicians in an emergency situation [4]. However, only a minority of medical students is sufficiently competent in performing PBLS [5, 6]. Previous research has also shown that the self-efficacy of medical students with regard to PBLS is inferior to their self-efficacy regarding adult BLS [4]. Self-efficacy refers to a person’s belief in his/her capability to organize and execute actions for the attainment of a particular goal [7, 8]. It is a predictor of behavior in that it influences the initiation of, devotion to, and perseverance in a certain action, despite challenges. As such, self-efficacy is context and task specific, as opposed to self-confidence, which is a more general, situation-independent personality trait, not significantly associated with future behavior [7, 8]. In the field of resuscitation, self-efficacy is clearly important, inasmuch as it relates to the confidence of providers to start and continue CPR when confronted with real patients in cardiac arrest. Thus, adequate PBLS training is crucial for medical students to improve their resuscitation skills and increase their self-efficacy.

At the time of our study, students following the bachelor curriculum of our medical school (Radboud University, Nijmegen, the Netherlands) only received adult basic life support (BLS) training. In the master phase, fifth-year medical students were trained in PBLS for the first time, just prior to their pediatric internship. This latter course was provided by pediatricians and involved a two-hour program, including a theoretical part (background information on pediatric resuscitation) and a practical part (PBLS training on a manikin). Expert-led training in small groups has been the routine for years, but with a growing clinical workload for specialists, both in our center and elsewhere, this type of training is becoming more difficult to realize. Peer-led BLS training can be an effective and valued alternative to teach medical students [9]. Peer teaching benefits medical students in that it offers education adjusted to their cognitive level and it creates a safe learning environment, because peer instructors are probably less threatening to students [10]. Nevertheless, no research has been conducted hitherto to evaluate the effect of peer teaching on the self-efficacy of medical students in the context of (P) BLS [9, 11]. It is known that healthcare professionals, who are skilled in resuscitation techniques, may fail to apply these techniques successfully, unless they have an adequately strong belief in their own capabilities [7]. Hence, there is a strong need for a curriculum, which enables medical students to gain a high level of self-efficacy.

Outside the context of (pediatric) resuscitation, there is some, though limited evidence that peer teaching promotes self-efficacy. In a study by Owens et al., peer instruction conduced to increased self-confidence regarding the performance of psychomotor nursing skills [12]. However, self-confidence is not the same as self-efficacy. Schunk demonstrated that peer models were better able to improve the self-efficacy of children in terms of learning cognitive skills than adult models could [13]. There are, in addition, two theory-based explanations that give credibility to the hypothesis that peer teaching stimulates self-efficacy. First, vicarious experiences or observational learning can generate self-efficacy in observers. Seeing others accomplishing a particular task makes them believe that they can also achieve success through perseverance; it motivates them to start performing that task [13, 14]. The most effective models for observational learning are demographically and psychosocially similar to, yet slightly more competent than the learners [7]. Near-peers neatly fulfil this description, as they are somewhat more advanced and competent than the students they teach, and they are ‘cognitively and socially congruent’ with their students [15]. Second, Artino stated that self-efficacy can be promoted by encouraging learners to set challenging and proximal goals, that is, targets within their range of abilities [14]. Ten Cate et al. basically recalled the same notion by referring to the ‘zone of proximal development’. This means that learning is thought to be most effective when the gap between what is known and what must be learned is just enough to incite study behavior in the learner. Since they are cognitively and socially congruent, near-peers are better able to ‘sense this zone’ and explore the needs and challenges of the students than expert teachers, who usually function on a different cognitive, social, and semantic level [15].

The primary aim of this study was to compare the PBLS-related self-efficacy of medical students who were trained by either expert instructors (pediatricians) or near-peer instructors. We also compared the skill performance of these two groups by assessing their pass rates on a simulated PBLS exam.

## Methods

### Instructors

Four pediatricians, working in our center (Radboud University Medical Center Amalia Children’s Hospital, Nijmegen, the Netherlands), took part as expert instructors. These pediatricians were very proficient and experienced in (teaching) PBLS. They were all certified instructors, not only locally, but also for national resuscitation courses developed by the European Resuscitation Council (ERC). All four pediatricians possessed nationally accredited university teaching qualifications and were all-round medical educators. They provided PBLS training to medical students, in the same way as described in this study, several times each year. They were familiar with the educational setting and medical curriculum.

Near-peer instructors were skilled and certified first aid instructors with at least three years of experience in teaching BLS and PBLS to students. They were all fifth or sixth-year medical students who had successfully completed their pediatric internship. They were randomly selected from the pool of student first aid instructors available in our center. All near-peer and expert instructors were invited to an additional instructor course, which contained background information on PBLS and didactic strategies involved in PBLS training.

### Participants

In our master curriculum, each month, a new group of approximately 30 fifth-year medical students follows a preparatory course for their pediatric internship. Within the time frame of this study, we were able to include all students of 7 groups that attended this course. Written informed consent was obtained from all students before study participation. The students were unaware of the aim of the study.

### Study design

**Fig. 1:**
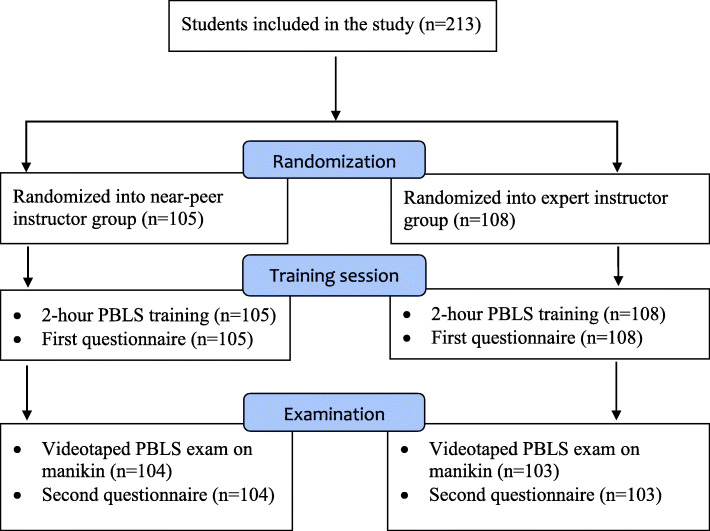
Flowchart

This study was a randomized controlled trial (Fig. 1). Blinding of the students was not achievable, considering our study design. During the 7-month study period, monthly PBLS training sessions were organized. An independent person was responsible for randomization. Half of the training sessions were led by expert instructors (expert instructor group, EIG), the other half by near-peer instructors (near-peer instructor group, NPIG). Apart from the intervention of interest (type of instructor), all educational interventions were identical in both groups. Thus, near-peer and expert instructors used the same teaching content and methods.

Before start of the training, background characteristics of the students were collected. Previous experience with PBLS was defined as any previous encounter with PBLS, either during training or in real life. Since competence in and self-efficacy regarding PBLS are importantly different than being proficient and self-efficacious in terms of adult BLS [4], we focused on previous PBLS exposure. The potential confounding effect of this characteristic was considered to be more important than that of earlier BLS experience. In general, all medical students attended at least one adult BLS course prior to participation in our study, for this is a mandatory component of their bachelor curriculum. Only a very small minority of medical students is confronted with the resuscitation of an adult in clinical practice.

PBLS training sessions lasted two hours. They took place in large classrooms at the authors’ institution. The instructor-to-student ratio was approximately 1:15. Training started with a lecture, during which background information on pediatric resuscitation and the step-by-step approach of the PBLS algorithm were taught, using an interactive teaching style. Students could ask questions throughout the presentation. Differences between infant and child resuscitation were highlighted. The presentation contained clear images taken from manuals and textbooks on PBLS as well as a few short instructional videos. Next, students assembled around the instructor, who provided a PBLS demonstration. This was followed by practical, hands-on training of PBLS skills on an infant and child manikin (SimBaby® and Resusci® Junior Basic, respectively, Laerdal Benelux, the Netherlands). Students rehearsed the technical and non-technical skills of the PBLS algorithm in small groups consisting of 2–3 students each. The instructor walked around, answered questions, provided additional skill demonstrations if necessary, and checked if all steps of the PBLS algorithm were performed adequately by the students. At the end, students had a final opportunity to discuss remaining uncertainties plenarily.

To assess students’ self-efficacy, a questionnaire was developed based on a validated scoring tool by Turner et al. [8]. Students completed this questionnaire directly after their training session. In the first part of the questionnaire, six visual analogue scales (VAS), ranging from 0 to 100, were used to assess self-efficacy regarding PBLS in general and compressions and ventilations in infant and child resuscitation in particular. In the second part of the questionnaire, students were asked to give a score (ranging from 0 to 100) for the following categories: theoretical education (6 questions), the quality of the feedback provided by the instructor (7 questions), and self-efficacy with respect to infant (7 questions) and child resuscitation (8 questions). At the end of the questionnaire, students were asked to evaluate the overall training session, giving it a mark between 1 (worst) and 10 (best).

A week after each training session, students completed a PBLS exam. This exam consisted of a standard PBLS scenario performed on a Resusci® Junior Basic manikin according to the algorithm described in the ERC guidelines [16], including 4 cycles of ventilations and compressions. We used the 2010 ERC guidelines as a reference, since our study took place just before the Dutch translation of the 2015 ERC guidelines was issued. Note that the PBLS algorithm was left unchanged in this latest update of the guidelines. All examinations were recorded on video. A random selection of approximately 50% of the videos was subsequently scored using a valid and reliable assessment instrument for PBLS [17]. In this scoring instrument, all items/steps of the PBLS algorithm, as issued by the ERC, are represented. Per item, 5, 10, 15, and 20 penalty points can be assigned to minor, moderate, substantial, and fatal errors, respectively. Clear and concise instructions are provided to guide the scoring process. To pass the exam, 15 penalty points or less are required. The intrarater reliability of this scoring instrument is substantial, with a weighted Cohen’s Kappa coefficient of 0.62 (95% CI: 0.45–0.81). The (single) person scoring the videotaped exams was blinded for the type of instructor. Following the exam, students completed a second VAS-based questionnaire on self-efficacy regarding pediatric resuscitation in general and compressions and ventilations in particular. This questionnaire was identical to the one completed after training as far as items on self-efficacy were concerned. Whereas the first questionnaire contained additional items on training aspects, this version had only one additional item on nervousness prior to the exam to gauge students’ sense of preparedness.

### Statistical analysis

Statistical analyses were performed using SPSS (SPSS, version 21.0.01, SPSS Inc., Chicago, Illinois, USA). The t-test and chi-square test were used to compare background characteristics. VAS-scores were analyzed by the independent samples t-test for parametric outcomes and by the Mann-Whitney U test for non-parametric outcomes. For analysis, all the subquestions in the second part of the questionnaire, belonging to one of the four main categories (theoretical education, feedback by the instructor, self-efficacy in infant resuscitation, and self-efficacy in child resuscitation), were combined, which resulted in four scores. Skewness and kurtosis were calculated to assess whether results were parametric or not. If so, the t-test was used to compare groups. If not, the Mann-Whitney U test was used. Categorical analyses were performed with the chi-square test. For all statistical tests, *p* < 0.05 was considered statistically significant.

## Results

### Background characteristics

Two hundred and thirteen students participated in this study: 105 in the NPIG and 108 in the EIG. Nine students did not provide their background characteristics. There were no significant differences between the two groups regarding sex, age, and previous experience with PBLS (Table 1).

**Table 1:** Background characteristics of the participating students

Characteristic	Near-peer instructor (*n* = 105)	Expert instructor (*n* = 108)	***p*** value
**Sex**			0.81
Male	32 (30.5%)	33 (30.6%)	
Female	71 (67.6%)	68 (63.0%)	
**Mean age (years)**	24.1	24.0	0.77
**PBLS experience**			0.72
None	80 (76.2%)	79 (73.1%)	
One course	21 (20.0%)	20 (18.5%)	
Real-life experience	1 (1.0%)	0 (0.0%)	
BLS instructor	1 (1.0%)	2 (1.9%)	
**Missing data**	2 (1.9%)	7 (6.5%)	

*BLS* basic life support

### Self-efficacy

On all six VAS, the NPIG scored significantly higher than the EIG (Table 2), indicating a higher self-efficacy regarding PBLS in general and compressions and ventilations on an infant and child in particular in the NPIG.

**Table 2:** VAS scores of self-efficacy regarding infant and pediatric resuscitation

	Near-peer instructor		Expert instructor		***p*** value
	Mean	95% CI	Mean	95% CI	
**Infant resuscitation in general**	65.58	62.98–68.28	55.28	51.58–58.98	< 0.001
**Compressions in infants**^a^	70.91	68.36–73.47	59.94	56.09–63.79	< 0.001
**Ventilations in infants**	64.44	61.46–67.42	51.58	47.63–55.53	< 0.001
**Child resuscitation in general**^a^	72.19	69.71–74.66	63.21	59.94–66.48	< 0.001
**Compressions in children**^a^	75.10	73.08–77.12	64.71	61.05–68.37	< 0.001
**Ventilations in children**^a^	71.76	69.51–74.00	62.54	58.86–66.23	< 0.001

^a^Non-parametric: Mann-Whitney U test

The results of the second part of the questionnaire showed considerable consistency with those of the first part. With a Cronbach’s alpha of 0.748 and 0.788 for infant and child BLS, respectively, our questionnaire had an acceptable internal validity. Near-peer-trained students scored significantly higher on self-efficacy relating to infant and child resuscitation than expert-trained students (Table 3).

**Table 3:** Marks for self-efficacy and quality of the training sessions

	Near-peer instructor		Expert instructor		***p*** value
	Mean	95% CI	Mean	95% CI	
**Self-efficacy in infant BLS**^a^	75.71	73.96–77.47	71.37	68.87–73.87	0.007
**Self-efficacy in child BLS**^a^	77.62	75.91–79.33	73.88	71.54–76.21	0.007
**Theoretical education**	82.29	79.56–85.03	79.72	77.25–82.19	0.35
**Quality of feedback**	85.78	83.79–87.77	86.70	84.90–88.50	0.58
**Overall mark**	8.08	7.95–8.21	8.09	7.97–8.22	0.72

^a^Non-parametric: Mann-Whitney-U-Test

*BLS* basic life support

### Appraisal of theoretical education and instructor

Training sessions were appreciated in both groups, with a mark of 8.08 and 8.09 in the NPIG and EIG, respectively (*p* = 0.72). There were no significant differences between the two groups regarding the appraisal of theoretical education and feedback provided by the instructor (Table 3).

### PBLS examination

Two hundred and seven students completed a PBLS exam (97.2%). Students in the NPIG reported a significantly higher self-efficacy regarding pediatric resuscitation in general and compressions and ventilations in particular than students in the EIG, with a mean difference of 6 points on a 0–100 scale. There was no difference in nervousness prior to the exam (*p* = 0.38) (Table 4).

**Table 4:** VAS scores of self-efficacy and nervousness for PBLS exam

	Near-peer instructor		Expert instructor		***p*** value
	Mean	95%CI	Mean	95%CI	
**Pediatric resuscitation in general**	71.41	69.35–73.46	65.39	62.84–67.93	< 0.001
**Compressions**	75.43	73.40–77.46	70.06	67.65–72.46	< 0.001
**Ventilations**	68.80	66.52–71.08	62.69	59.31–66.07	< 0.001
**Nervousness**	27.87	23.61–32.16	31.89	27.58–36.20	0.38

Half (102, of which 52 in the NPIG and 50 in the EIG) of the videotaped PBLS exams were assessed with the validated assessment instrument. Pass rates were similar in both groups (NPIG 67.3% vs. EIG 62.0%, *p* = 0.58). The mean number of penalty points was 15.67 in the NPIG and 16.50 in the EIG (*p* = 0.69) (Table 5).

**Table 5:** Skill performance on the PBLS examination

	Near-peer instructor (*n* = 52)	Expert instructor (*n* = 50)	***p*** value
**Penalty points**	15.67	16.50	0.69
**Result**	35 passed (67.3%)	31 passed (62.0%)	0.58

## Discussion

Our findings suggest that medical students trained by near-peer instructors develop a higher level of self-efficacy regarding all aspects of infant and child BLS than students trained by expert instructors. Near-peers are seemingly able to ensure that medical students feel properly prepared for BLS in infants and children. Focusing on self-efficacy is important. It is a predictor of behavior in that it influences the initiation and continued performance of (resuscitative) efforts, despite challenges and setbacks [7]. Self-efficacy is believed to be of particular importance in the context of resuscitation, because it influences the development of and access to the associated knowledge and skills [7]. Unfortunately, there are no studies available on the association between students’ self-efficacy and their performance of PBLS skills. In general, the self-efficacy of students regarding PBLS is much lower than their self-efficacy regarding adult BLS [4], though it increases with training [18]. It appears that the correlation between self-efficacy and quality of pediatric resuscitation requires further elucidation [19, 20]. In a study, in which consultants and trainee pediatricians and anesthesiologists scored their self-efficacy for pediatric resuscitation skills before taking an unannounced simulated resuscitation test and objective structured clinical examination (OSCE) of chest compressions and bag and mask ventilation, self-efficacy correlated moderately with the quality of global performance on the simulation test, but not with the OSCE scores, nor was quality of individual skills during the simulation related to self-efficacy [19]. Plant et al. found a significant, positive correlation between pediatric residents’ self-efficacy in situation awareness and environment management and overall performance of crew resource management skills [20]. These authors suggested that, in a specific context, self-efficacy, as a form of self-assessment, may be informative with regard to performance.

It appears that near-peer instructors are at least as capable as expert instructors in teaching basic resuscitation skills to medical students. The fact that near-peer-instructed medical students passed their PBLS exam in a rate comparable to that of students trained by experts shows that near-peer teaching can be equally effective. These findings seem to support the possibility of (partially) replacing busy and costly specialists by near-peer instructors in PBLS courses.

Our results correspond with those of Hughes et al.*,* who also found no difference in pass rate between peer-trained and expert-trained medical students on a BLS exam [11]. A study by Perkins et al. even showed a higher pass rate when students were taught by peers compared to clinical staff [9]. A peer-led training program improved the performance and retention of BLS skills of pharmacy students [21]. Recently, a randomized controlled trial (RCT), conducted in Syria, also showed that peer-led training was as effective as professional-led training in delivering theoretical BLS knowledge and practical BLS skills to medical students [22]. Peer-led training resulted in student satisfaction, and peer-trained students indicated that they were more at ease and experienced greater motivation, interaction, and enthusiasm than professional-trained students. Professionals were, on the other hand, better able to answer difficult questions than peers. The authors of this report emphasized the value of peer education for BLS in countries with limited resources. The number of participants included in this study was relatively small (64 students) and BLS skills were assessed with a non-validated performance checklist. German researchers also performed an RCT, including 1087 secondary school students, to investigate whether hands-on BLS mass training provided by peer instructors was non-inferior to training offered by professionals [23]. Although this study could not demonstrate the non-inferiority of peer-led training compared to professional-led training – using a non-inferiority margin of 5% – due to an inadequate sample size, the pass rates of both groups for the practical BLS examination were very similar (40.3% vs. 41.0%, respectively), corroborating the effectiveness of peers as life support instructors. Again, the assessment tool used in this study was not validated. The comparability in BLS skills between peer-trained and professional-trained participants in the Syrian and German trials is especially notable, considering the fact that peers were relatively inexperienced in both studies; they merely attended a single/short instructor course prior to the study. In addition, the extensive single-centre experience and literature review described by Harvey et al. clearly support the beneficial effects and excellent outcomes of peer involvement in BLS training for healthcare students [24]. However, none of these studies investigated self-efficacy of the students as outcome measure.

Ten Cate and Durning described 12 distinct reasons for a broader application of peer teaching [10]. This type of education can be beneficial to learners, because it is tailored to their cognitive level. It may also lower the threshold to ask questions. Experts may fail to understand the problems that students encounter while learning certain skills, due to a significant cognitive and communicative gap. On the other hand, peer teaching may also favour the teachers themselves. It enables peer educators to develop leadership skills and didactic qualities, which are unequivocally important for their future career as medical specialists. Recently, an article, containing twelve tips on how a peer-led medical education society can be set up and run, was published [25].

Three students participating in our study were BLS instructors. These students were not excluded from analysis, because their small number and more or less even distribution over both study groups made it highly unlikely that their exclusion would have altered our results. Moreover, being a BLS instructor was not a predefined exclusion criterion, so exclusion would have constituted a post-hoc modification. One student in the NPIG and five students in the EIG did not perform the PBLS exam. We were not informed about the reason for their absence. In general, the main reason for such absence is intercurrent illness of the student or a family member. For the analysis of skill performance, we used a random selection of approximately 50% of the videotaped PBLS exams. Thus, the drop outs did not affect these results.

There are some limitations to this study. Two expert instructors were not able to attend the additional instructor course. Although well-motivated, they were too busy with their clinical work at that time. This may have resulted in a different instruction style compared to the other trainers. However, these expert instructors already had years of experience in teaching PBLS to medical students. Our study had a rather early endpoint, which was deliberately chosen. As the medical students fan out for their internships shortly after this course, later endpoints would have caused major loss to follow up. Another shortcoming was the fact that we did not perform a pretest to determine baseline self-efficacy and skills prior to the training sessions. Since both study groups consisted of same-year medical students with an equally small amount of experience in PBLS, it is unlikely that baseline self-efficacy and competence were importantly different between groups. Finally, as said before, students could not be blinded in this study design.

## Conclusions

Our results point towards the fact that near-peer-trained medical students can develop a higher level of PBLS-related self-efficacy than expert-trained students. PBLS skill performance was similar in both training groups. Based on our findings, some previous work [12, 13], and the abovementioned theoretical considerations, the hypothesis that (near-)peer teaching has an intrinsic, positive impact on self-efficacy may gain credibility. Future studies are needed to support this hypothesis. Also, it remains to be determined whether the seemingly higher self-efficacy following peer-led pediatric resuscitation training is retained in the long run, whether our results can be extrapolated to different healthcare professionals and other forms of life support training, and whether increased self-efficacy actually translates into improved performance of real-life (pediatric) resuscitation.

## Data Availability

All data generated or analyzed during this study are included in this published article.

## Abbreviations

BLS: Basic life support
CPR: Cardiopulmonary resuscitation
EIG: Expert instructor group
ERC: European Resuscitation Council
OSCE: Objective structured clinical examination
PBLS: Pediatric basic life support
NPIG: Near-peer instructor group
RCT: Randomized controlled trial
VAS: Visual analogue scale
